# The complete genomic sequence of an in vivo low replicating BLV strain

**DOI:** 10.1186/1743-422X-6-120

**Published:** 2009-08-03

**Authors:** Syamalima Dube, Lynn Abbott, Dipak K Dube, Guillermina Dolcini, Silvina Gutierrez, Carolina Ceriani, Marcela Juliarena, Jorge Ferrer, Raisa Perzova, Bernard J Poiesz

**Affiliations:** 1 Department of Medicine, Upstate Medical University, Syracuse, New York 13210, USA; 2 Universidad Nacional del Centro de la Provincia de Buenos Aires, Facultad de Ciencias Veterinarias, Tandil, Argentina; 3 Consejo Nacional de Investigaciones Científicas y Técnicas (CONICET), Argentina; 4 Comisión de Investigaciones Científicas y Técnicas de la Provincia de Buenos Aires (CIC), Argentina; 5 Comparative Leukemia and Retroviruses Unit, New Bolton Center, University of Pennsylvania, Kennett Square, Pennsylvania 19348, USA

## Abstract

DNA was extracted from lamb lymphocytes that were infected in vivo with a BLV strain after inoculation with the peripheral blood mononuclear cells from a persistently sero-indeterminate, low viral load, BLV-infected Holstein cow (No. 41) from Argentina. The DNA was PCR amplified with a series of overlapping primers encompassing the entire BLV proviral DNA. The amplified BLV ARG 41 DNA was cloned, sequenced, and compared phylogenetically to other BLV sequences including an in vivo high replicating strain (BLV ARG 38) from the same herd in Argentina. Characterization of BLV ARG 41's deduced proteins and its relationship to other members of the PTLV/BLV genus of retroviruses are discussed.

## Background

Bovine leukemia virus (BLV) is an infectious agent of cattle that can cause B-lymphocytic lymphoma/leukemia and benign disorders that, directly or indirectly, have a financial impact on the cattle industry [1-3]. It is estimated that more than 10 and 30% of the dairy and beef cattle in the United States and Argentina, respectively, are infected with BLV [1,2,4]. BLV, together with the primate T-cell leukemia lymphoma viruses (PTLV), form a separate genus of retroviruses that exhibit in vivo lymphotropism and are characterized by the transforming property of a unique virus regulatory protein, Tax, that can transactivate both viral and cellular genes [[5] and [6]]. A sizeable minority (5–20%) of cattle or primates infected with BLV or PTLV, respectively, either take a long time (>2 years) or never fully seroconvert [7-9]. Detection of infection in seronegative or seroindeterminate hosts requires PCR analyses of peripheral blood mononuclear cells (PBMC) for viral DNA; such analyses usually indicate a relatively low viral DNA copy number compared to high titer seropositive subjects [10]. RNA-PCR assays for viral RNA in the plasma and/or PBMC from such low DNA copy subjects are negative, while high titer seropositives have copy numbers ranging from 0 to 10,000 copies per ml [5]. The reason(s) for these differences in seroconversion and peripheral blood viral loads among BLV and PTLV infected hosts are unknown, but certainly could be due in part to genetic differences among viral strains. Previously, we published the full length sequence of BLV ARG 38, a viral strain obtained from a high titer seropositive, high viral load Holstein cow from a commercial herd of dairy cattle maintained near the Facultad de Ciencias Veterinarieas de Tandil, Argentina (FCV-UNCP-BA) [11]. Herein, we describe the sequence of BLV Arg 41, a BLV isolate obtained from another cow from that same herd that was persistently seroindeterminate and had persistently low BLV viral DNA loads.

## Results

### BLV Arg 41 isolation

Cows 38 and 41 were members of a Holstein dairy herd in TandilBalcarce, Argentina that was routinely monitored over a many year period for BLV infection using serologic assays for anti BLV antibodies and PCR assays of PBMC for BLV DNA. Both cows remained clinically healthy over eight years of observation, but cow 38 had a persistent lymphocytosis (PL). Cow 38 was found to have a high viral load (>10,000 copies of BLV **pol **DNA per μg of PBMC DNA) and rapidly (<3 months) seroconverted with high titer (range 200 to 800) of antibodies to both BLV p24 gag (titer ~200) and gp51 env (titer ~800) proteins. These high viral DNA loads and high titers of anti-BLV antibodies persisted over 8 years of observation. The complete genomic sequence of the BLV strain infecting cow 38 (BLV ARG-38) has been previously published [11].

When first sampled in October 1995, cow 41 had been low titer (50–100) antibodies to gp51, no antibodies to p24, and was also PCR negative for BLV. In March, 1996, it developed low titer antibodies to BLV p24 (10). Since then, it has had persistent low titer antibodies to gp51 but has remained seronegative to p24 and, hence, would be deemed to have indeterminate seroreactivity to BLV antigens. It was first found to be PCR positive for BLV DNA in October, 1996, with a viral load of 160 copies of BLV DNA per μg of PBMC DNA. Since then, it has been persistently PCR positive, but with viral loads ranging from 5 to 10 copies of BLV DNA per μg of PBMC DNA. The viral strain infecting cow 41 is referred to as BLV ARG 41.

Because the initial copy number of BLV ARG 41 in PBMC was so low, the cloning and sequencing of PCR amplified BLV DNA proved to be difficult. Hence, we attempted to isolate the BLV ARG 41 strain by inoculating a lamb with 130 ml of heparinized blood from cow 41. This lamb (p12) rapidly seroconverted (<3 months) with persistent high titer antibody to both BLV p24 gag and gp51 env antigens. The BLV DNA copy number in p12 PBMC has been persistently >5,000 copies per μg of cellular DNA.

Using DNA from post infection p12 PBMC, PCR amplification and Southern blot hybridization were successful for each of the BLV primer pair/probe groups utilized. The complete sequence of BLV ARG 41 was obtained from these amplified products (Gen Bank Accession No. FJ914764). No variability was observed among the many overlapping clones sequenced from each of the regions amplified, indicating that the p12 cells were infected with one unique strain of BLV.

**Figure 1:**
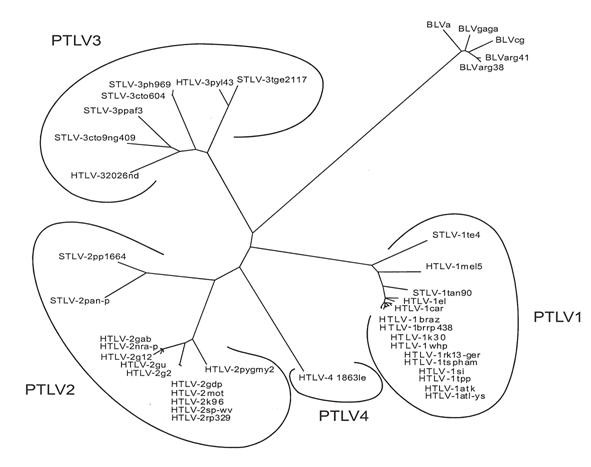
**Phylogenetic tree comparing 662 bases of *pol *DNA from human (HTLV) and simian (STLV), PTLV strains and five BLV strains**. Bootstrap values for all except the most terminal branches are greater than 90%. The bootstrap value for the branch that contains BLV Arg 41 and BLV Arg 38 is 100%. The bar at the lower left indicates the length of a 10% distance between sequences. As can be seen, the PTLV segregate into four distinct species, while the BLV strains constitute a single species.

Comparative analyses indicate that BLV ARG 41 is approximately 98.9% homologous to BLV ARG 38, 95.6% homologous to BLV A from Australia, 96.4% homologous to BLV GAGA from Belgium, and 96.4% homologous to BLV CG from Japan. Phylogenetic analyses (Fig 1) confirm that BLV ARG 41 and BLV ARG 38 are most homologous to each other.

**Figure 2:**
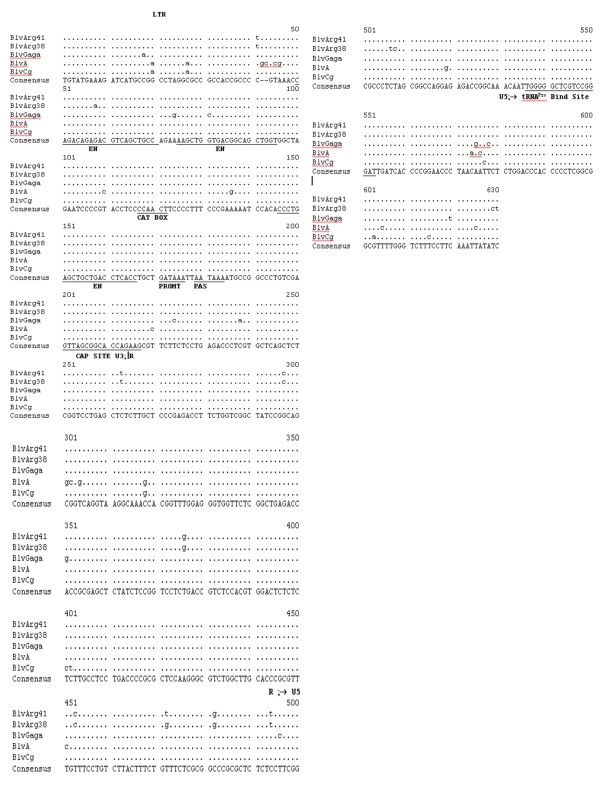
**Long terminal repeat nucleic acid sequences of BLV Arg 41 compared to 4 other BLV strains including BLV Arg 38, BLV GAGA, BLVA and BLVCG **[17,25,26]**The consenus sequence is shown at the bottom of the alignment**. A bullet indicates homology with the consensus sequence, while the nucleic acid substitutions are as indicated. The U3, R, and U5 regions of the LTR are as indicated. The three enhancer (EN) regions, the CAT BOX and GATAA (PROMT) box promoters of RNA transcription, the polyadenylation site (PAS), the CAP site and the tRNA proline primer binding sites are as shown.

Comparison of the LTR of BLV ARG 41 with that of the other full-length BLV sequences is shown in Fig. 2. It contains the RNA transcription promoter and enhancer elements, NF-KB binding site between the second and third enhancers, cyclic AMP response elements (CRE) and E box motifs within the enhancers, glucocorticoid response element 5' to the third enhancer, PU box, interferon regulatory factor binding site, polyadenylation signal, REX response element and the tRNA proline primer binding site typical of BLV. BLV ARG 38 and BLV ARG 41 have identical sequences in these functional regions except for the sequences in the CRE contained in the first enhancer element (AGACGTCA for BLV Arg 41 and AAACGTCA for BLV Arg 38). It is unknown what effect this change might have on viral transcription. However, other experimental changes in the BLV CRE have been shown to alter transcriptional activity [12]. The only other small differences between the two sequences occur in regions of the LTR that are not believed to be of functional significance. Both strains have the same number and location of CpG methylation sites. Hence, there is only one difference in the LTRs of BLV ARG 38 and BLV ARG 41 that could explain the *in vivo *differences in serologic reactivity and viral load observed in their respective bovine hosts.

**Figure 3:**
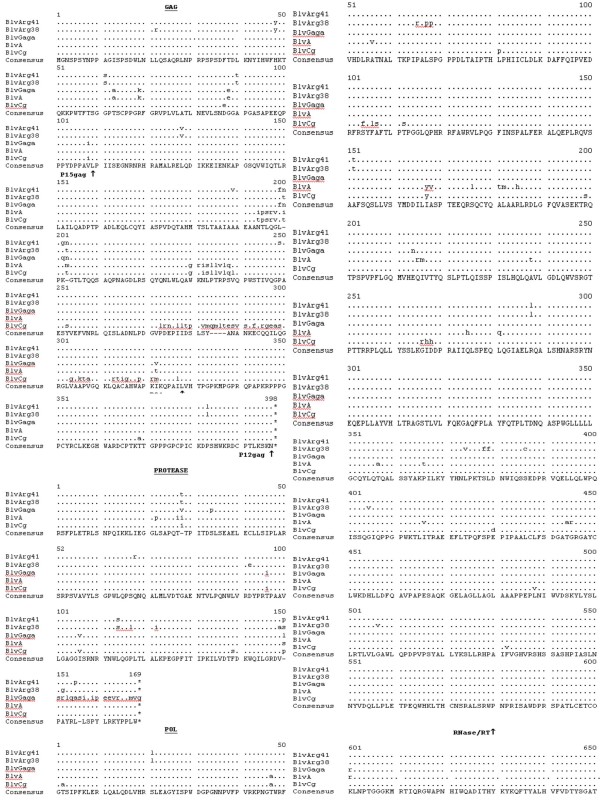
**Deduced amino acid sequences of the various proteins of BLV Arg 41 compared to four other published BLV strains: BLV Arg 38, BLV GAGA, BLV A and BLV CG**. The consensus sequences are shown at the bottom. A dash is shown in the consensus sequences in areas of nonagreement. The bullets show areas of homology with the consensus sequence, while the amino substitutions are as indicated and deleted amino acids are indicated by the dash symbol. The ends of the various proteins are indicated by the up arrow. Stop codons are indicated by asterisks. Only a portion of the BLV A Tax is shown, and the rest is indicated by the an wavy lines. In the Env proteins the N-linked glycosylation sites are shown in bold, while the neutralizing domains (ND), the transmembrane hydrophobic region (TMHR), and various immunostimulatory epitopes are as shown. In the GIV protein the two putative cellular protease cleavage sites are indicated by an inverted triangle and the amino acid myb-like motif (MYB) and the arginine-rich nucleus targeting RNA-binding region (ARNTRB) are shown.

**Figure 4:**
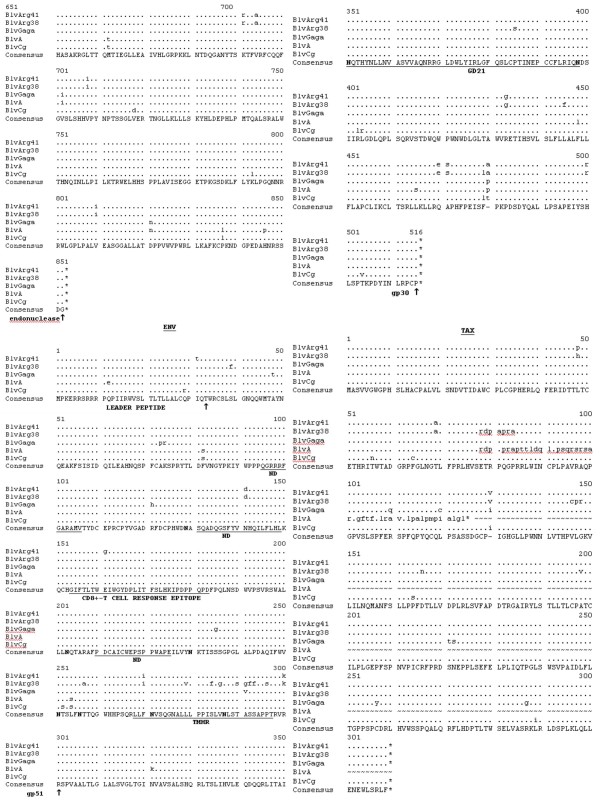
**Deduced amino acid sequences of the various proteins of BLV Arg 41 compared to four other published BLV strains: BLV Arg 38, BLV GAGA, BLV A and BLV CG**. The consensus sequences are shown at the bottom. A dash is shown in the consensus sequences in areas of nonagreement. The bullets show areas of homology with the consensus sequence, while the amino substitutions are as indicated and deleted amino acids are indicated by the dash symbol. The ends of the various proteins are indicated by the up arrow. Stop codons are indicated by asterisks. Only a portion of the BLV A Tax is shown, and the rest is indicated by the an wavy lines. In the Env proteins the N-linked glycosylation sites are shown in bold, while the neutralizing domains (ND), the transmembrane hydrophobic region (TMHR), and various immunostimulatory epitopes are as shown. In the GIV protein the two putative cellular protease cleavage sites are indicated by an inverted triangle and the amino acid myb-like motif (MYB) and the arginine-rich nucleus targeting RNA-binding region (ARNTRB) are shown.

While the BLV **gag **region is highly conserved, it is apparent (Fig. 3) that there are two different peptide sequences preferred for p15 and p24. However, there are only minor differences between BLV ARG 38 and BLV ARG 41. One potentially functional difference could be the amino acid change P140S (position 249 in GAG in Fig 3) in the BLV Arg 41 p24 gag protein. This change lies in the major homology region of all retroviruses and, wherein, other mutations have been shown to affect BLV infectivity [13]. There were no differences in the two p24 epitopes responsible for anti-BLV T-cell recognition in BLV infected animals [14]. The BLV CG strain is markedly divergent over the last 40 amino acids of its p24 protein. Whether this is a real observation or the result of sequencing error (Fig. 4) and whether these changes would have functional consequences are unknown. However, because this is a highly conserved, immunodominant region in the BLV/PTLV genus, it seems likely that such a change would have significant biological consequences [[15] and [16]].

The deduced protease proteins among the five BLV strains (Fig. 3) were also highly conserved, except for the fact that the published sequence of BLV GAGA contains an insertion that causes a frame shift at amino acid (aa) 150 and eliminates a stop codon at aa 169 (Fig. 3 &4). Again, it is highly likely that this difference may be due to a sequencing error in BLV GAGA. While BLV ARG 38 has four unique aa changes relative to all the other four BLV strains, BLV ARG 41 has one. It is unknown whether these changes could affect protease function.

The deduced RNase, reverse transcriptase, and integrase amino acids encoded by the *pol *gene of all five BLV strains are highly conserved (Fig. 3 and 4), with there being two slightly different peptide sequences among the five strains. There are nine unique amino acids in the RNase/RT of BLV ARG 38 relative to all of the other BLV strains, including BLV ARG41. There are no differences between the integrases of BLV ARG 38 and BLV ARG 41.

The env leader peptides and the transmembrane gp30 env proteins of all five strains are also highly conserved, as are the gp51 surface env proteins of four of the strains (Fig. 4). BLV ARG 38, however, demonstrates significant divergence at the carboxyl terminus of its gp51 env protein. This is the transmembrane hydrophobic region of gp51, believed to be responsible for anchoring the surface env protein in the viral membrane [17]. Hydrophobicity plots (data not shown) indicate that this region of BLV ARG 38 would be more hydrophobic than BLV ARG 41 and the other three strains and, theoretically, more stably embedded in the viral envelope.

Save for minor differences, all N-linked glycosylation sites and neutralizing domains in both env proteins are conserved. The following important functional domains are also identical: the putative cell surface receptor binding sites and neutralizing domains on the surface gp51 env protein; the peptide region in gp51 that induces a CD8^+ ^cytotoxic T-cell response in the host cow; the highly immunogenic epitope GD21 that is conserved in all members of the PTLV/BLV genus; the tetrapeptide WAPE (aa 222–225 in Fig. 4) that has been shown to be critical for infection; and the amino acids P and D (aa 210 and 211 in Fig. 4) that have been shown to induce T-helper proliferative responses in cattle [15-17]. There is one unique mutation, E161G, in the CD8^+ ^T-cell response epitope of the gp51 env of BLV ARG 41 that theoretically could alter the stimulation of the anti-BLV CD8^+ ^T-cell response [6].

**Figure 5:**
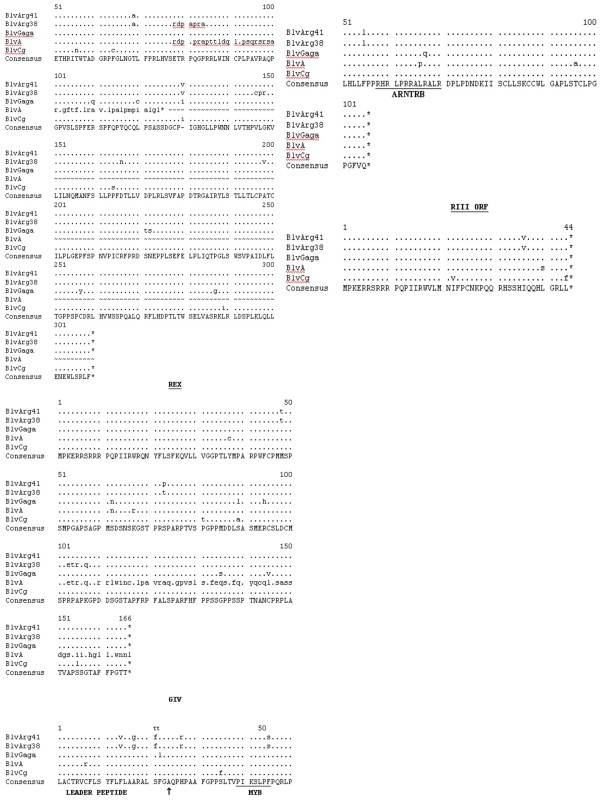
**Deduced amino acid sequences of the various proteins of BLV Arg 41 compared to four other published BLV strains: BLV Arg 38, BLV GAGA, BLV A and BLV CG**. The consensus sequences are shown at the bottom. A dash is shown in the consensus sequences in areas of nonagreement. The bullets show areas of homology with the consensus sequence, while the amino substitutions are as indicated and deleted amino acids are indicated by the dash symbol. The ends of the various proteins are indicated by the up arrow. Stop codons are indicated by asterisks. Only a portion of the BLV A Tax is shown, and the rest is indicated by the an wavy lines. In the Env proteins the N-linked glycosylation sites are shown in bold, while the neutralizing domains (ND), the transmembrane hydrophobic region (TMHR), and various immunostimulatory epitopes are as shown. In the GIV protein the two putative cellular protease cleavage sites are indicated by an inverted triangle and the amino acid myb-like motif (MYB) and the arginine-rich nucleus targeting RNA-binding region (ARNTRB) are shown.

**Figure 6:**
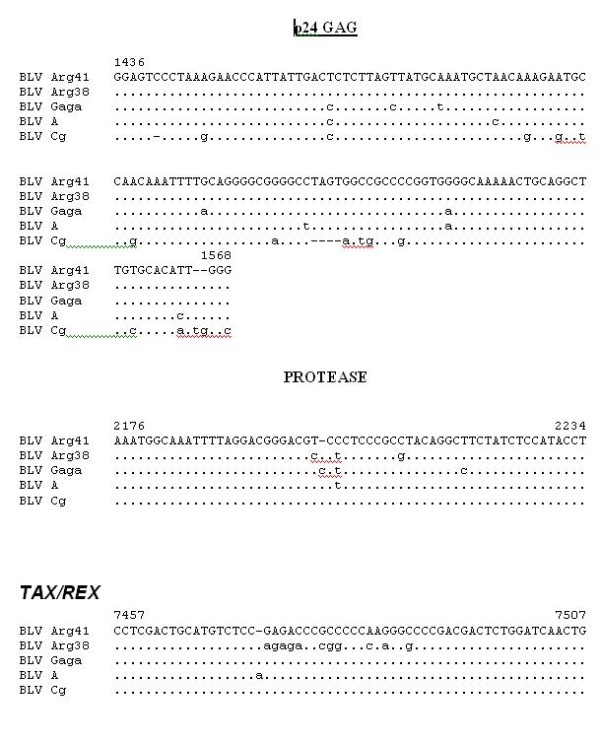
**Nucleic acid aligments of regions of suspected errors among published BLV sequences**. These include: 1) a deleted C (base 1441), and TAGT (bases 1521–1524) in the BLV CG p24 gag sequence; 2) an inserted C (base 2202) in the BLV GAGA protease sequence; and 3) an inserted A (base 7475) in the BLV A **tax/rex **sequence.

Comparison of the five deduced BLV Tax and Rex proteins again demonstrates what is probably the result of sequencing errors in BLVA (Fig. 4, 5, and 6). With that noted, there are two different peptide sequences evident in aa 78–84 of the Tax protein, with BLV ARG 41 being different from BLV ARG 38. Because this area has been shown to be critical for Tax transactivation, these differences could result in variable viral replication and/or host cell transformation [18]. All told, there are 13 different aa in the BLV ARG 41 vs BLV ARG 38 Tax proteins. There are five aa differences between the BLV ARG 41 and BLV ARG 38 Rex proteins.

The deduced amino acids from two peptides, GIV and RIII ORF, which are translated from alternatively spliced mRNA's known to be expressed in BLV-infected cells, are also shown in Fig. 5. The expression of GIV, has been associated with PL in infected cattle [19]. Cow 38 exhibits PL and its GIV protein is quite divergent from BLV Gaga, BLV A, and BLV Cg; however, it is identical to the BLV ARG 41 sequence and cow 41 did not exhibit PL.

## Discussion

BLV is a member of a genus of retroviruses that cause a variety of malignant and autoimmune diseases in cattle, humans, and nonhuman primates. While much is known regarding the clinical sequelae of BLV infection in domesticated cattle, little is known of its genetic diversity, epidemiology, and disease association among bovids around the planet. Because the human retroviruses, HTLV-1, 2, 3, and 4 share a common ancestor with BLV, understanding this genetic diversity and its biological implications is of interest to human as well as veterinary medicine.

Continued epidemiological studies indicate that there are two distinct chronic infection states among BLV infected cattle [10]. One, characterized by a high viral DNA load and antiviral antibody titer, is associated with a higher frequency of peripheral blood lymphocytosis and B-lymphocytic leukemia/lymphoma. The other is characterized by a low viral DNA load, lower antibody titers and a more favorable clinical prognosis.

There are several possible explanations for the differences in the infection profiles observed among BLV infected cattle. These include biologic differences among strains of BLV and/or among the bovine hosts. Recently, we have published data regarding the correlation of various BoLA genotypes found in cattle with the development of either the high viral load or low viral load infectious profiles [[20,21], Juliarena M.A., Ceriani C., Dube S., Poli M., Gutierrez S., Dolcini G., Sala L., Poiesz B., and Esteban E.] Further characterization of the bovine leukemia virus (BLV) infection profile named low proviral load (LPL), submitted). These data suggest that genetic differences among cattle influence the replication of BLV among infected hosts and the development of leukemia/lymphoma.

The publication of full length BLV sequences from both a high [11] and a low (this study) proviral load infected cow should allow for future comparisons of BLV infected cattle to ascertain whether differences in viral strains could also explain the observed infectivity patterns. These comparisons should also elucidate the biologic importance of genetic differences observed in functional or structural regions of the BLV genome.

## Materials and methods

### Cow 41

PBMC were obtained from a Holstein (Holando-Argentino) dairy cow (No. 41). Cow 41 had been proven to be infected with BLV by PCR serology assays, including ELISA and Western blot assays for antibodies to BLV p24 and gp51 env proteins, as previously described [9,10,22]. Cow 41 remains in good health and never developed PL. It's antibody titers against the above BLV antigens and it's BLV proviral load were monitored episodically over eight years. One hundred thirty ml of peripheral blood from cow 41 were used to subcutaneously inoculate a lamb (p12), which was monitored for BLV infection, as above. This lamb became infected with the BLV Arg 41 strain.

### Nucleic Acid and Amino Acid Studies

DNA was organically extracted from either cow 41 or lamb p12 PBMC and amplified via PCR using overlapping primer pairs that encompass the entire BLV genome, as previously described [11]. The amplified products were detected by Southern blot hybridization using ^32^P-labeled oligonucleotide probes located between the flanking primers. Amplified specific products were cloned into a TA cloning vector (Invitrogen, San Diego, CA), and sequenced using an automated sequencer (Applied Biosystems, Foster City, CA). Several clones were sequenced for each primer pair, and sequences were obtained for each strand of DNA. Both nucleic acid and deduced amino-acid sequences were aligned [23]. Six hundred and sixty two bases of **pol **sequence from the BLV, HTLV, and STLV strains shown in Figure 1 were analyzed via the neighbor-joining technique, as previously described [5]. One hundred boot-strap replications were performed. These sequences were derived from the following GenBank accession numbers: D13784; AY563953; L10341; AF259264; L03561; AF139170; AF042071; U19949; M86840; J02029; S74562; AY563954; L02534; Y14365; AF326583; AF326584; AF139382; M10060; L11456; Y13051; X89270; AF412314; L020734; AF074965; Y14570; U90557; AF391797; AF391796; Y07616; AY217650; DQ020493; AF517775; AY818421; AY818422; AY222339; Z46900; AF074966; K02120; M35242; M35239; and AF257515.

Hydrophobicity plots of the gp51 env proteins from the five BLV strains shown in Figure 3 were generated using the Network Protein Sequence Analysis program [24].

## Competing interests

The authors declare that they have no competing interests.

## Authors' contributions

SD and LA conducted most of the PCR amplification and subsequent sequencing of BLV ARG41. DD participated in data analysis. GD, SG, CC, MJ conducted the serologic and quantitative PCR assays, and the isolation and culture of BLV ARG41 in Argentina. JF helped organize the experiments in Argentina. RP participated in phylogenelotic analyses. BP oversaw experiments in Syracuse, and participated in data analysis. All authors participated in manuscript preparation and experimental design. All authors read and approved the final manuscript.
